# A Systematic Review and Integrated Bioinformatic Analysis on the Gene Expression of Cumulus-Oocyte Complex Following In Vitro Maturation

**DOI:** 10.21315/mjms-12-2024-948

**Published:** 2025-06-30

**Authors:** Marjanu Hikmah Elias, Nurzahidah Zainal, Siti Nabillah Abdul Rahman, Zulazmi Sutaji, Mohd Faizal Ahmad, Nur Fariha Mohd Manzor

**Affiliations:** 1 Faculty of Medicine and Health Sciences, Universiti Sains Islam Malaysia, Negeri Sembilan, Malaysia; 2 Department of Obstetrics and Gynaecology, Faculty of Medicine, Universiti Kebangsaan Malaysia, Kuala Lumpur, Malaysia

**Keywords:** infertility, cumulus cells, in vitro maturation, differentially expressed gene, molecular pathway, gene ontology

## Abstract

Infertility, a rising global concern, is frequently caused by genetic causes, making modern reproductive methods like in vitro fertilisation (IVF) and in vitro maturation (IVM) necessary for successful conception. Nonetheless, little is known about the molecular pathways causing infertility. This study conducted a systematic review following PRISMA guidelines, aiming to comprehensively elucidate the gene expression profiles of cumulus cells following IVM and their implications for reproductive outcomes. A thorough literature search was conducted across multiple databases, employing a combination of keywords related to cumulus cells, IVM, and gene expression. For this systematic review, screening processes identified four clinical human studies meeting inclusion criteria, published between 2013 and 2022. The studies used qPCR and RNA sequencing to compare gene expression in cumulus cells pre- and post-IVM or between IVF and IVM patients. Despite variations in sample sizes and methodologies, 22 differentially expressed genes (DEGs) were identified, with 10 common DEGs between IVF and IVM matured cumulus-oocyte complexes. Protein-protein interaction network analysis revealed a complex molecular network associated with cumulus cell function and oocyte maturation. Clustering analysis identified a significant cluster enriched in genes involved in the epidermal growth factor receptor signalling pathway and cell membrane dynamics. Gene ontology and pathway enrichment analysis highlighted the involvement of DEGs in cell-cell signalling, signal transduction, and ovarian steroidogenesis pathways. The findings emphasise the importance of understanding molecular mechanisms in infertility and provide valuable insights for optimising assisted reproductive technologies. Future research should focus on validating these findings and exploring potential therapeutic targets for improving reproductive outcomes.

## Introduction

Assisted reproductive technologies have emerged as a critical component in treating infertility issues and providing hope to infertile couples in recent times. However, a lower rate of success in a certain couple leads to a multicycle IVF treatment becoming the primary factor, causing the couple to discontinue their treatment (1). In vitro maturation (IVM) is one of these technologies that has shown promise in improving IVF outcomes by maturing oocytes outside the conventional in vivo setting. By allowing oocytes to mature in a controlled laboratory environment, IVM modifies traditional in vitro fertilisation (IVF) techniques by obviating the necessity for hormonal stimulation of the ovaries (2).

The main objective of IVM is to maximise oocytes’ developmental potential by letting them mature in vitro without hormonal stimulation to the patients, thus providing a more natural setting. This method offers a simplified and economical approach to fertility treatment while minimising the risks related to hormone stimulation (3). It is a patient-friendly alternative. IVM is also crucial for patients who, despite receiving hormonal stimulation, are unable to develop mature eggs, such as those with polycystic ovarian syndrome patients (4). Thus, exploring the complex molecular and genetic mechanisms behind IVM is essential for improving our knowledge of its effects on oocyte quality and subsequent embryo quality.

Oocyte quality is at the forefront of in vitro fertilisation (IVF) research since it is crucial to inadequate fertilisation and embryonic development (5). Several investigations have examined the relationship between embryo quality and gene expression profiles of the granulosa cell population surrounding the oocyte, known as cumulus cells, after IVF (6) and IVM (7). Essential responsibilities in directing oocyte maturation are played by the cumulus cells surrounding the oocyte in the cumulus-oocyte complex (COC). Cumulus cells act as metabolic guardians, supplying vital nutrients and energy substrates required for oocyte development (8). Concurrently, they produce steroid hormones, including oestrogen, which affect the oocyte’s growth and the surrounding cumulus cells through autocrine signalling (9).

A unique communication network known as gap junctions links the oocyte and cumulus cells and allows chemicals and signalling compounds to be exchanged (10). The synchronised maturation of the encapsulated oocyte and cumulus cells depends on this two-way communication. By generating extracellular matrix components, cumulus cells also aid in the growth of the cumulus mass, a crucial stage in releasing oocytes during ovulation (11).

Cumulus cells orchestrate the complex regulatory processes necessary for oocyte maturation at the molecular level by activating signalling pathways such as cyclic AMP (cAMP) (12) and mitogen-activated protein kinase (MAPK) (10). Cumulus cells generate antioxidants as protectors against oxidative stress, preserving the genomic integrity of the oocyte as it matures. During oocyte maturation, cumulus cells shape the molecular landscape by influencing gene expression in the oocyte (13). Understanding the various roles and processes of cumulus cells is crucial for improving IVM and other assisted reproductive technologies (14). It also has the potential to enhance and modify the results of reproduction.

Nevertheless, most of the molecular role that cumulus cells play in promoting oocyte maturation comes from research conducted on animals of different species (7, 15, 16). Therefore, it is essential to ascertain the gene expression of human cumulus cells to ensure that the mechanisms described in animal studies correspond with the functions of human cumulus cells. In doing this integrated bioinformatic analysis and systematic review, our goal is to thoroughly assess the body of research on gene expression patterns in the cumulus cells of infertile women after IVM and to identify the candidate genes associated with oocyte maturation in IVM. This integrative approach will provide a holistic understanding of the genetic landscape governing the success of IVM procedures among infertile women. Therefore, the objective of this study is to comprehensively elucidate the gene expression profiles of cumulus cells following IVM and their implications for reproductive outcomes.

## Methods

This review has been officially registered in PROSPERO (No. CRD42024514185).

### Search Strategy

The PRISMA guideline was used to conduct the systematic review. A thorough literature search was conducted using ProQuest Central, EBSCOhost, ScienceDirect, PubMed, and Scopus. Relevant research articles released until 7 February 2024 were located. Keywords such as “cumulus cell,” “in vitro maturation,” and “gene expression” were taken from the Medical Subject Heading (MeSH). MeSH terms from the Cochrane Library were used to generate synonyms for keywords. Through the evaluation of gathered review articles, further text terms were discovered. The following sets of keywords were combined as part of the search strategy: i) “cumulus cell,” “granulosa cell,” or “cumulus”; ii) “in vitro maturation,” “IVF,” or “in vitro fertilisation”; iii) “in vitro fertilisation,” or “in vitro fertilisation”; iv) “gene expression,” or profiling; or v) transcriptome OR transcriptomic; with (“AND”) serving as the connector. The bibliographies of the retrieved papers yielded further references.

### Inclusion Criteria

All cross-sectional, case-control, and prospective observational studies were included in investigating the differentially expressed genes (DEGs) in the cumulus cells of infertile women following IVM treatment. However, to ensure data homogeneity, only clinical human studies identifying DEGs in the post-IVM cumulus cells of infertile women were included.

### Exclusion Criteria

Editorials, case reports, conference proceedings, and narrative review articles were excluded since they did not contain primary data. Excluded studies included in silico, in vitro, in vivo, and animal models. Intervention trials that used any extra drugs or supplements in the IVM media were excluded from consideration. Studies that obtained their RNA from oocytes were disqualified. Studies that did not provide further information, links to external sources, or a list of DEGs in their paper were excluded from consideration. Excluded from consideration are studies that included participants with endometriosis and polycystic ovary syndrome, as well as people with chronic diseases, including diabetes and cancer.

### Screening of Articles for Eligibility

**Figure 1:**
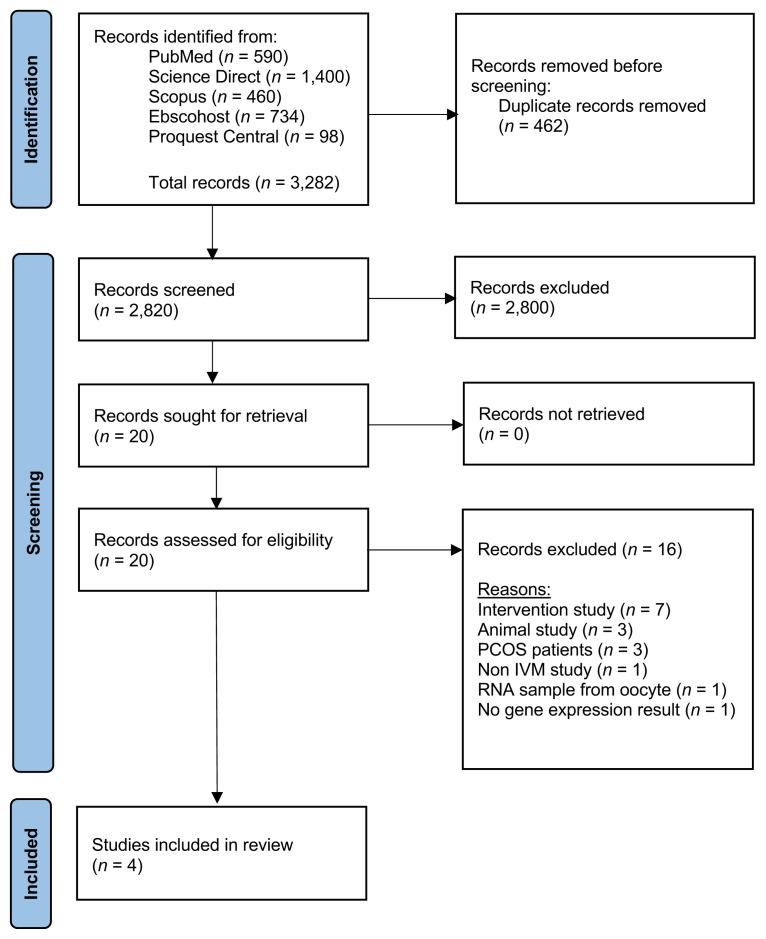
The systematic review’s PRISMA flow diagram was used for study selection

The articles that were collected from all resources underwent three stages of screening. In the first step, duplicates were eliminated, and all articles with irrelevant titles were disqualified. In the second phase, the abstracts of the remaining papers were reviewed, and those that did not fit the inclusion criteria were eliminated. Lastly, the full texts of the remaining articles were thoroughly examined. In this third stage, every article that did not fit the requirements for inclusion was eliminated. The phases of screening, choosing, and extracting data involved every author. The PRISMA flow diagram summarising the article assortment procedure and the grounds for item removal is displayed in Figure 1.

### Data Extraction

The studies that met the inclusion requirements had their data extracted. The process of extracting the data involves all the authors. The data-gathering process was standardised using a data collection form, and each data extraction process was carried out separately. When there were conflicts, they were discussed, and the majority decided. Reasons for rejection were noted in the records. The information gathered is as follows: i) author name; ii) year of publication; iii) article title; iv) country; v) study design; vi) sample size; vii) gene expression method; viii) list of upregulated genes; ix) list of downregulated genes.

### Study Quality Assessment

Using the Joanna Briggs Institute critical assessment tools, NFMM, NZ, NAH, and ZS independently assessed each paper’s study quality. MHE and MFA verified the validity of the study’s findings. Studies with an overall score of less than 50% were classified as low quality (high risk of bias), those with an overall score between 50 and 69% as moderate quality (moderate risk of bias), and those with a score of more than 69% as high quality (low risk of bias).

### Protein-protein Interaction (PPI) Network, Clustering, and Visualisation

All selected DEGs from the Venn diagram analysis were pooled and analysed through PPI functional-enrichment analysis via STRING software (https://string-db.org/) to identify the PPI network (17). Results from STRING were exported into Cytoscape software (http://www.cytoscape.org/) to visualise molecular interaction networks and integrate gene expression profiles of the DEGs (18). The Cytoscape MCODE plug-in was implemented to perform module analysis of the target network and protein clustering. The module-selection criteria included degree cut-off = 2, node score cut-off = 0.2, node density cut-off = 0.1, K-score = 2, and max depth = 100. The list of genes in each cluster was subsequently analysed independently using the Database for Annotation, Visualization, and Integrated Discovery (DAVID) software to distinguish significantly enriched gene ontology.

### Gene Ontology (GO) and Pathway Enrichment Analysis

All the genes in each cluster were analysed using DAVID to discover the GO that exhibited significant functional-annotation enrichment related to cervical cancer pathogenesis (19, 20). The involvement of genes in the pathway linked to cervical cancer was identified based on the Kyoto Encyclopedia of Genes and Genomes (KEGG) pathway.

## Results

A total of 3,282 possibly pertinent studies from the five databases were found using the keywords. Titles were used to eliminate 462 duplicates, and the abstracts of the remaining 2,820 were reviewed. Two thousand eight hundred papers were eliminated after the abstract was evaluated. Then, all the documents from the other 20 papers were obtained. Following a comprehensive examination of the entire text, 16 papers that did not fit the inclusion and exclusion criteria were eliminated. Ultimately, a total of four studies were chosen for the systematic review. These four studies were published between 2013 and 2022. Homogenised sampling was employed to make sure that sample bias was avoided by closely adhering to the exact inclusion and exclusion criteria. The sample size ranged from 5 to 72, and only papers containing gene expression results were selected. The summary of the features of the included studies is presented in Table 1.

### Patient Recruitment and Sample Collection Details

One hundred participants from four studies were included in this systematic review, aged 13 to 39. All studies collected cumulus cells for gene expression analysis. The gene expression analysis was done by using qPCR and RNA sequencing. Cadenas et al. (21) and Guzman et al. (24) compare the gene expression of cumulus cells before and after IVM. Barzilay et al. compared the gene expression of cumulus cells between IVF and IVM patients, while Coticchio et al. (22) did both comparisons of pre- vs post-IVM and IVF vs IVM cumulus cells. Nevertheless, the demographic profile of the patients was not further described in any of the studies.

### Study Quality

Supplementary 1 contains a comprehensive quality assessment of the included studies. Two studies with a moderate risk of bias and four high quality studies with a low risk of bias comprised the included studies.

### DEGs in Cumulus Cells Following IVM

**Figure 2:**
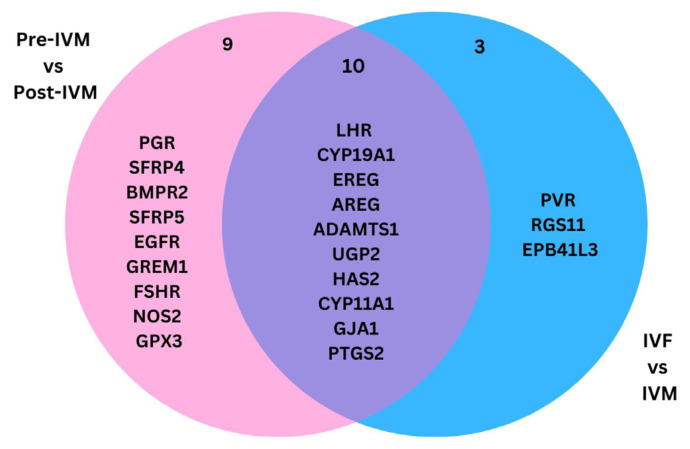
Venn diagram of the DEGs from post-IVF vs post-IVM and pre-IVM vs post-IVM

After excluding duplicates, 22 DEGs were extracted from all the selected studies. The DEGs reported are retrieved from two different analyses of pre-IVM against post-IVM and IVF against IVM. A comparison of gene expression between pre- and post-IVM shows that 19 genes were differentially regulated, while 13 genes were differentially regulated when gene expression in IVF is compared to IVM. Interestingly, using Bioinformatics and Evolutionary Genomics software (25), 10 common DEGs are shared between pre-IVM against post-IVM and IVF against IVM. Figure 2 shows the Venn diagram of the DEGs reported in all selected studies.

### PPI Network and DEGs Clustering

**Figure 3:**
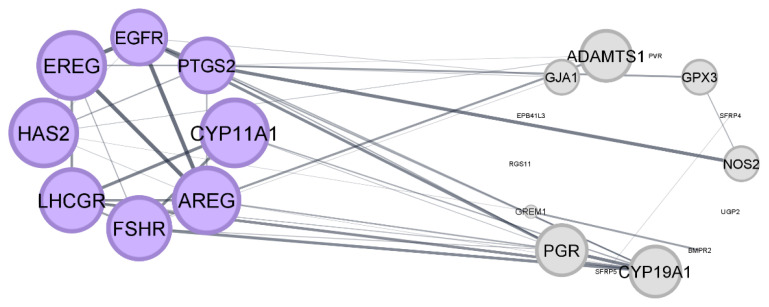
PPI network and clustering of the DEGs collected from the selected studies

PPI network and modular analysis were used to identify the critical candidate genes and biological pathways. A PPI network complex comprising 22 nodes and 42 edges, with an average local clustering coefficient of 0.569 and a PPI enrichment *P-*value of < 1.0e-16, was formed by filtering 22 proteins from the extracted DEGs. The network’s data was moved from STRING to Cytoscape to visualise the molecular interaction networks. From the PPI network complex, only one cluster was found by applying the Molecular Complex Detection method (MCODE). The significant cluster produced by the PPI network complex derived from the DEGs in cumulus cells after IVM is depicted in Figure 3. According to the functional-annotation clustering, Cluster 1 has 17 edges and 8 nodes (score = 4.857).

### GO and Molecular Pathways

The GO and pathway enrichment analysis showed that most of the DEGs reported by the selected studies are primarily located in the extracellular space and cell surface. Table 2 shows the gene ontology of all the differentially expressed genes. Based on their biological process of the GO, these DEGs are involved in the cell-cell signalling and signal transduction processes, including the regulation of cell proliferation and some of the genes involved in the ovarian steroidogenesis pathway.

After clustering with MCODE, a single cluster was found. Table 3 shows the functional-annotation clustering of the DEGs in Cluster 1. Four DEGs in Cluster 1 were primarily located at the clathrin-coated endocytic vesicle membrane. Based on their biological function, these DEGs in Cluster 1 are involved in the epidermal growth factor receptor signalling pathway, particularly the ERBB2-EGFR signalling pathway. Their molecular functions include ATPase binding.

## Discussion

Different gene expression patterns were found in cumulus cells before and after in vitro fertilisation (IVM), as well as between IVM and traditional IVF methods, according to the results of the chosen research. A total of 22 DEGs were found through comparative analysis despite differences in sample sizes and patient demographics among the studies. Notably, in both comparisons, it was discovered that 10 DEGs were frequently dysregulated, providing insight into putative molecular markers linked to IVM. The expression of these 10 genes differs between pre-IVM and post-IVM, indicating that the COC maturation occurs. However, these genes are also differentially expressed between IVM and IVF groups, suggesting there is dysregulation of gene expression in in vitro matured COC compared to in vivo matured COC. The 10 genes include Luteinising Hormone/Choriogonadotropin Receptor (*LHCGR*), *CYP19A1, CYP11A1*, epiregulin (*EREG*), Amphiregulin (*AREG*), von Willebrand factor-cleaving protease (*ADAMST*), UDP-Glucose Pyrophosphorylase 2 (*UGP2*), Hyaluronan Synthase 2 (*HAS2*), Gap Junction Protein Alpha 1 (*GJA1*) and Prostaglandin-Endoperoxide Synthase 2 (*PTGS2*).

LHCGR is a G protein-coupled receptor predominantly expressed on granulosa and theca cells within the ovarian follicles. Luteinising hormone (LH) activation triggers a cascade of intracellular signalling events crucial for cumulus cell expansion and subsequent oocyte maturation (26). Once oocyte maturation is achieved, LHCGR undergoes major suppression in response to increased levels of human chorionic gonadotropin (hCG) during ovulation. In this systematic review, the expression of LHCGR in cumulus cells shows significant downregulation after IVM (22), showing the maturation of the oocyte. Oocyte maturation involves complex interactions between the oocyte and surrounding cumulus cells, and changes in oocyte developmental competence may affect LHCGR expression levels (27). However, the downregulation of LHCGR in COC among IVM matured oocytes does not reduce as low as in vivo matured oocytes (22). The differences in LHCGR expression between IVM and in vivo matured COC can be due to the variations in hormone concentrations and signalling dynamics that may influence the expression and regulation of LHCGR.

As LHCGR is required for the activation of adenylyl cyclase via G proteins by the binding of LH through the luteinising hormone signalling pathway (BP_GO:0042700), the expression of AREG and EREG will be upregulated accordingly (28, 29). This will subsequently induce the resumption of meiosis and the oocyte release during ovulation. HCG can also regulate AREG expression in theca cells, thus conforming to the higher expression level of AREG as LHCGR upregulates since LHCGR acts as both LH and hCG receptors (28). In this review, AREG, EREG, and LHCGR genes were downregulated in vitro compared to in vivo, providing insights into the interaction between these genes and their correlation to oocyte maturation and cumulus cell expansion.

On the other hand, HAS2 was found to be upregulated in post-IVM cumulus cells (22, 24). HAS2 synthesises hyaluronic acid, a large polysaccharide molecule, which contributes to forming the cumulus cell-extracellular matrix surrounding the oocyte (30). This matrix provides structural support to the oocyte and cumulus cell expansion. It also facilitates communication between the cumulus cells and the oocyte during maturation. This systematic review showed a significantly higher expression of HAS2 among IVF-mature COC compared to IVM-mature COC (22, 24). In IVF COCs, the expression of HAS2 is supported by young donors’ adipose-derived stem cells (ASCs), enhancing cumulus cell migration and, subsequently, maturation rates (31). Thus, the supportive environment in the ovary provided by ASCs contributes to the higher HAS2 expression in IVF COCs, highlighting the importance of the cumulus cells’ microenvironment in influencing gene expression levels.

Furthermore, additional investigation using the PPI network and modular analysis revealed the significance of potential genes and biological pathways in the maturation of oocytes and the function of cumulus cells. The emergence of a discrete cluster in the PPI network mainly correlated with the extracellular domain, the cell surface, and cell-cell communication mechanisms, highlighted the complex interactions between cumulus cells and their microenvironment throughout follicular maturation.

Of particular interest, DEGs within Cluster 1 were implicated in the epidermal growth factor receptor (EGFR) signalling pathways and the ErbB signalling pathway. Their localisation to clathrin-coated endocytic vesicle membranes and molecular functions, including ATPase binding, further elucidated their roles in cellular signalling and endocytic processes crucial for oocyte maturation and follicular development.

AREG, an epidermal growth factor (EGF) family member, is mainly expressed in the placenta and functions in inducing oocyte maturation (32). EREG, on the other hand, is also a member of the EGF family, which encodes a protein-ligand of EGFR and functions in activating RAS and ERK1/2 required for oocyte maturation (33). Following gonadotropin surge, the expression of AREG and EREG in the follicular fluid of pre-ovulatory follicles upregulates (34) to serve as a mediator of LH-induced EGF network (BP_GO:0038134) by binding to EGFR, a protein receptor regulated by GDF9 and BMP15, thus stimulating the expression of downstream genes which coordinate COC expansion, oocyte meiotic maturation, and ovulation (35). Activated EGFR signalling induces the activation of maturation-promoting factor (MPF) by disrupting intercellular communication, primarily through the gap junctions Cx43 (36), which turns off the interaction between cGMP and phosphodiesterase 3A (PDE3A). Hence, activated PDE3A can degrade cyclic adenosine monophosphate (cAMP), consequently inhibiting PKAI, which acts to suppress cdc25. Therefore, the release of cdc25 from inhibition facilitates the activation of maturation-promoting factors (37).

The role of AREG in oocyte maturation is supported by a study on AREG knockout mice, where it was reported that the absence of this gene led to decreased oocyte meiotic resumption, cumulus expansion, and ovulation (38). In another study, AREG was expressed significantly higher in pregnant women than in non-pregnant women, providing a conclusive understanding of the positive correlation between AREG and good-quality oocytes (39). However, the downregulation of AREG and EREG in IVM was reported to be an indicator of lower pregnancy outcomes and live birth rates (40).

The removal of immature cumulus-oocyte complexes from the follicle during IVM is said to be attributable to a lower quality maturation due to the removal of roots, leading to undesired elimination of some essential elements of the granulosa and theca cells, which concurrently influences the oocyte maturation (40). In IVM, culturing medium is commonly supplemented with FSH to mediate nuclear maturation, hCG to enhance the maturation of oocytes, and LH to favour early embryonic development (41) and activate the EGF/EGFR network (42). However, in some studies, adding hCG and LH was unnecessary for developing immature oocytes, which also explains the downregulation of the AREG and EREG genes in the in vitro technique. Apart from that, environmental pressure exerted on the oocytes during IVM is one factor leading to a lower quality of oocytes (43).

On the other hand, the rise of LH level leads to the high expression of Progesterone Receptor (PGR), a steroid receptor transcription factor essential for follicular rupture (44). As LHCGR on mural granulosa cells is activated, adenylate cyclase (AC) will be stimulated, thus increasing cAMP. A high level of cAMP leads to the activation of protein kinase A (PKA), which induces the expression of PGR, whereas low LH levels and impaired cAMP have been proven to cause reduced expression of PGR. In ovulation, PGR regulates ADAMTS1, cathepsin L, and ADAM8 proteases, which are crucial in cumulus expansion. To this date, only the role of ADAMTS1 has been clearly understood, which mediates the neo-morphogenesis of the ovulating follicle wall (45). However, PGR does not directly regulate oocyte quality and meiosis resumption. In another study, it was found that in PGR knockout mice, ovulation did not occur spontaneously; instead, oocyte retrieval was needed before the ovulation to allow the oocytes to reach the blastocyst stage (46), which justifies the upregulation of PGR IVM compared to in vivo in this review.

Limitations of this systematic review include the heterogeneity in study methodologies, sample sizes, and patient demographics among the included studies, which may limit the generalizability of findings. Furthermore, the reliance on data from observational studies may introduce biases and confounding factors that could influence the interpretation of results. Despite these limitations, the strengths of this systematic review lie in its rigorous adherence to the PRISMA guidelines, comprehensive literature search across multiple databases, and thorough screening process. Additionally, using standardised data extraction methods and quality assessment tools enhances the reliability and validity of the findings. Integrating PPI network analysis and GO enrichment analysis provides valuable insights into the molecular mechanisms underlying cumulus cell function and oocyte maturation. Thus, this systematic review is a valuable resource for researchers and clinicians in reproductive medicine, offering essential implications for future research and clinical practice.

## Conclusion

The findings from this systematic review provide a comprehensive overview of the molecular landscape of cumulus cells following IVM. PPI network analysis elucidated complex molecular networks associated with cumulus cell function and oocyte maturation while clustering analysis identified a significant cluster enriched in genes involved in the EGFR signalling pathway and cell membrane dynamics. Furthermore, GO and pathway enrichment analysis highlighted the involvement of DEGs in critical biological processes such as cell-cell signalling, signal transduction, and ovarian steroidogenesis pathways. These findings have important implications for optimising assisted reproductive techniques. However, further research is warranted to validate these findings and elucidate the precise mechanisms underlying the observed changes in gene expression in cumulus cells during IVM.

## Supplementary Material

**Supplementary 1:** Joanna Briggs Institute (JBI) critical appraisal checklist for case control studies

Author (Year)	Type of checklist	1	2	3	4	5	6	7	8	9	10	Total	Percentage
Barzilay (2014)	JBI critical appraisal checklist for case control studies	Y	Y	Y	Y	Y	Y	N	Y	Y	UN	8	80
Cadenas (2022)	JBI critical appraisal checklist for case control studies	Y	Y	Y	Y	Y	N	NA	Y	Y	Y	8	80
Coticchio (2013)	JBI critical appraisal checklist for case control studies	Y	Y	UN	Y	Y	Y	N	Y	Y	UN	7	70
Guzman(2013)	JBI critical appraisal checklist for case control studies	Y	Y	Y	Y	Y	Y	Y	Y	Y	Y	10	100

JBI critical appraisal checklist for case control studies		Yes	No	Unclear	Not applicable
1.	Were the groups comparable other than the presence of disease in cases or the absence of disease in controls?	□	□	□	□
2.	Were cases and controls matched appropriately?	□	□	□	□
3.	Were the same criteria used for identification of cases and controls?	□	□	□	□
4.	Was exposure measured in a standard, valid and reliable way?	□	□	□	□
5.	Was exposure measured in the same way for cases and controls?	□	□	□	□
6.	Were confounding factors identified?	□	□	□	□
7.	Were strategies to deal with confounding factors stated?	□	□	□	□
8.	Were outcomes assessed in a standard, valid and reliable way for cases and controls?	□	□	□	□
9.	Was the exposure period of interest long enough to be meaningful?	□	□	□	□
10.	Was appropriate statistical analysis used?	□	□	□	□

## Figures and Tables

**Table 1:** Characteristics summary of the included studies

Author (year)	Country	Study design	Sample size (*n*)	Method	Upregulated genes	Downregulated gene
Cadenas et al. (2022) (21)	Denmark	Case-control	Pre-IVM = 24 Post-IVM = 48	qPCR	BMPR2 SMAD3 SMAD5 ALK6	-
Coticchio et al. (2017) (22)	Italy	Case-control	IVF = 2 Pre-IVM = 2 Post-IVM= 2	qPCR	Pre vs Post-IVM: AREG EREG HAS2 SFRP5 CYP19A1 PTGS2 UGP2 ADAMTS1 IVM vs IVF: HAS2 CYP11A1 CYP19A1 PTGS2 ADAMTS1	Pre vs Post-IVM: SFRP4 LHCGR CYP11A1 NOS2 GPX3 GJA1 GREM1 BMPR2 IVM vs IVF: LHCGR AREG EREG UGP2 GJA1
Barzilay et al. (2014) (23)	Israel	Case-control	IVF = 3 IVM = 2	RNAseq and qPCR	RGS11 EPB41L3	PVR
Guzman et al. (2013) (24)	Belgium	Case-control	Pre-IVM = 5 Post-IVM = 12	qPCR	HAS2 FSHR EGFR PGR	-

**Table 2:** Gene ontology of all the differentially expressed genes

Term	Description	Genes	*P*-value
CC_GO:0005886	Plasma membrane	GJA1, BMPR2, NOS2, FSHR, EPB41L3, HAS2, PGR, RGS11, PVR, EGFR, EREG	0.020
CC_GO:0005615	Extracellular space	GREM1, SFRP4, BMPR2, GPX3, SFRP5, AREG, PVR, EGFR, EREG	3.20E-04
CC_GO:0009986	Cell surface	GREM1, SFRP4, BMPR2, FSHR, AREG, PVR, EGFR	2.69E-05
CC_GO:0005789	Endoplasmic reticulum membrane	GJA1, HAS2, PTGS2, CYP19A1, AREG, EGFR	0.003
CC_GO:0005887	Integral component of plasma membrane	GJA1, BMPR2, FSHR, HAS2, EGFR, EREG	0.011
BP_GO:0007267	Cell-cell signalling	GREM1, GJA1, PGR, AREG, EREG	8.50E-05
BP_GO:0008285	Negative regulation of cell proliferation	SFRP4, ADAMTS1, SFRP5, PTGS2, EREG	0.001
BP_GO:0008284	Positive regulation of cell proliferation	GREM1, HAS2, AREG, EGFR, EREG	0.002
BP_GO:0007165	Signal transduction	GREM1, GJA1, SFRP5, PGR, EGFR	0.041
MF_GO:0030297	Transmembrane receptor protein tyrosine kinase activator activity	GREM1, AREG, EGFR, EREG	9.61E-07
MF_GO:0020037	Heme binding	NOS2, CYP11A1, PTGS2, CYP19A1	5.51E-04
hsa04913	Ovarian steroidogenesis	CYP11A1, FSHR, PTGS2, CYP19A1	1.03E-04

**Table 3:** Functional-annotation clustering of the DEGs in Cluster 1

Term	Description	Genes	*P*-value
CC_GO:0030669	Clathrin-coated endocytic vesicle membrane	AREG, EGFR, EREG	1.13E-04
BP_GO:0038134	ERBB2-EGFR signalling pathway	AREG, EGFR, EREG	1.48E-06
BP_GO:0007173	Epidermal growth factor receptor signalling pathway	AREG, EGFR, EREG	6.71E-05
BP_GO:0042700	Luteinising hormone signalling pathway	LHCGR, EREG	5.14E-04
MF_GO:0051117	ATPase binding	LHCGR, PGR, EGFR	2.01E-04
hsa04012	ErbB signalling pathway	AREG, EGFR, EREG	9.37E-04
